# Editorial: Extremophiles: Microbial genomics and taxogenomics

**DOI:** 10.3389/fmicb.2022.984632

**Published:** 2022-08-02

**Authors:** Rafael R. de la Haba, André Antunes, Brian P. Hedlund

**Affiliations:** ^1^Department of Microbiology and Parasitology, Faculty of Pharmacy, University of Sevilla, Sevilla, Spain; ^2^State Key Laboratory of Lunar and Planetary Sciences, Macau University of Science and Technology, Taipa, Macau SAR, China; ^3^China National Space Administration (CNSA), Macau Center for Space Exploration and Science, Macau, Macau SAR, China; ^4^School of Life Sciences, University of Nevada, Las Vegas, NV, United States; ^5^Nevada Institute of Personalized Medicine, University of Nevada, Las Vegas, NV, United States

**Keywords:** extremophiles, taxonomy, genomics, metagenomics, adaptation, Space Microbiology, extremozymes, biodiversity

## Introduction

Extreme habitats exist across the globe and account for most of our planet's habitable zone by volume (Gold, 1992; Charette and Smith, 2010). They vary widely from a physical-chemical perspective as they include diverse types of extremes, such as temperature, pH, salinity, radiation, pressure, water activity, and nutrient availability. Organisms that thrive under conditions that are adverse or lethal for most organisms are called “extremophiles” (Brock, 1969).

All three domains of life are represented in each type of extreme environment. However, the vast majority of extremophiles are bacteria and archaea (Rampelotto, 2013), which is not surprising considering their remarkable diversity and adaptability. The discovery, study, and classification of novel microbial extremophiles is of enormous interest, given their potential biotechnological applications (Corral et al., 2019) and the ever-increasing expectation to find life on other planets, where conditions might be similar to those of extreme environments on Earth (Gómez et al., 2012).

Our understanding of extremophiles on Earth is rooted in studies of pure cultures, but has greatly expanded over the last few decades through both intellectual and technological advancements in molecular ecology, including several next-generation DNA sequencing and bioinformatics applications, which have evolved rapidly over the last decade. Recent developments increasing the fidelity and lowering the cost of both short- and long-read sequencing technologies hold promise for dense, accurate, and inexpensive DNA sequencing studies focused on both microbial pure cultures and microbial communities (Slatko et al., 2018). In parallel, increases in computing power per Moore's law (Moore, 1965) and the development of new bioinformatic tools, machine learning algorithms, and data mining approaches now provide the necessary means to deal with such a huge amount of data (Gauthier et al., 2019; Goodswen et al., 2021).

Extreme environments possess a larger than expected microbial diversity, considering the harshness of the conditions that extremophiles need to cope with (Shu and Huang, 2022). Many efforts have been made to characterize and describe prokaryotic and eukaryotic extremophiles and their metabolic functions within these habitats using culture-dependent approaches (de la Haba et al., 2011; Nogi, 2011; Yumoto et al., 2011; Sorokin et al., 2014; Ventosa et al., 2014; Deming, 2019; Johnson and Aguilera, 2019; Nienow, 2019; Santos and Antón, 2019; Topçuoglu and Holden, 2019; Sood et al., 2021). Nevertheless, estimates using 16S rRNA gene sequences and fixed similarity cutoffs suggest that around 80 % of microbial genera remain uncultured in non-human-associated environments (Lloyd et al., 2018). Similarly, analysis of shotgun metagenomic data estimated that yet-uncultivated taxa account for ~85 % of the phylogenetic diversity of prokaryotes (Nayfach et al., 2021). Thus, most of the diversity and functions of extremophiles remain poorly understood. Fortunately, high-performance DNA sequencing and computation-based analysis now allow the routine recovery of high-quality metagenome-assembled genomes (MAGs) (Yang et al., 2021) and single-cell genomes (Rinke et al., 2013), in addition to genomes of cultured microorganisms (Whitman et al., 2015). These approaches finally allow us to describe high-quality genomes from extremophilic “microbial dark matter,” which serve as a foundation to guide both field and lab studies of the ecology and physiology of these organisms within the context of the environments and communities they inhabit.

The use of genomic data of both cultured and uncultured extremophiles is also valuable to infer evolutionary relationships and to establish a robust and accurate classification system (Parks et al., 2022), and a formalized code of nomenclature based on genome sequences as nomenclatural types will soon be available (Murray et al., 2020). Most existing taxa of extremophiles were proposed based on classical but increasingly old-fashioned polyphasic studies that included 16S rRNA gene phylogenetic analyses and wet-lab DNA-DNA hybridization approaches, which have limited resolution and suffer from problems with accuracy and reproducibility among laboratories, respectively. These approaches have therefore been overtaken by phylogenomic studies and *in silico* genome relatedness indices (Chun and Rainey, 2014; Jain et al., 2018). Therefore, the current taxonomy of extremophiles needs to be assessed using genomic information. The ever-increasing access to genomic databases and the ever-decreasing cost of next-generation sequencing technologies and computing makes this a particularly timely Research Topic and grants us an exceptional opportunity to address this issue.

The present Research Topic includes 25 manuscripts spanning the discovery and characterization of new microbial extremophiles, the biotechnological applications of their enzymes, the mechanisms of adaptation to harsh environments, their metabolic pathways and potential ecological roles, their evolution, systematics and biodiversity, as well as exciting research on Space Microbiology and the persistence of extremophiles in low-biomass spacecraft assembly cleanrooms. The Research Topic call was well received, with a total of 185 contributing authors from 18 different countries around the world (Australia, China, Czech Republic, Denmark, Egypt, France, Germany, India, Japan, Mexico, Norway, Poland, Russia, Saudi Arabia, Spain, South Korea, United Kingdom, and United States), which reflects global interest on extremophiles. At the time of writing, over 150,000 views of this Research Topic have been recorded. The high impact of this Research Topic has lead us to establish a community series and make a second call for manuscripts to be submitted to Community Series-Extremophiles: Microbial Genomics and Taxogenomics, Volume II.

## Discovery of new extremophiles and extremozymes

Considering that only a small fraction of extremophilic taxa possess a cultured representative, it has never been more important to encourage the scientific community to isolate and describe not-yet-cultured extremophiles. New high-throughput cultivation approaches, referred as “culturomics” (Lagier et al., 2012), coupled with focused, genome-guided cultivation efforts (Tyson et al., 2005; Buessecker et al., 2022), will broaden our knowledge about extremophiles, as a major complement to metagenomics. This collection of articles contributes to this aim with the description and characterization of new extremophilic isolates belonging to both Bacteria and Archaea, as well as a novel viral genus.

Three papers from the Duroch group describe new taxa of thermophilic cyanobacteria in the family *Leptolyngbiaceae*, including two new species, *Thermoleptolyngbya sichuanensis* (Tang et al.) and *Leptodesmis sichuanensis* (Tang, Du et al.), and a new genus and species, *Leptothermofonsia sichuanensis* (Tang, Shah et al.). As suggested by their species names, all three were isolated from hot springs in Sichuan Province, China; the morphological, physiological, genomic, and taxonomic data presented in this collection represent a significant expansion of the systematics of the family and of thermophilic cyanobacteria in general. The Research Topic also includes the proposal by Slobodkina et al. of a new member of the class *Archaeoglobi*, at that time represented by only eight validly published species names (Parte et al., 2020). The new species *Archaeoglobus neptunius* was isolated from a deep-sea hydrothermal vent on the Mid-Atlantic Ridge and possesses a lithoautotrophic mode of nutrition, although it is also able to grow chemoorganotrophically. This article points to polyphyletic origin of the species currently grouped into the genus *Archaeoglobus*, necessitating a future review of its taxonomic status.

In addition to thermophiles, new psychrotolerant bacterial species were proposed within this collection. The paper from Králová et al. describes two new psychrotolerant species of *Flavobacterium* isolated from Antarctica, namely, “*Flavobacterium flabelliforme*” and “*Flavobacterium geliluteum*”, including three and five strains, respectively. As might be expected, both taxa harbor several cold-inducible or cold-adaptation related genes, but surprisingly they also accommodate numerous prophages and displayed a multidrug-resistant phenotype. Genome analysis revealed the presence of unknown biosynthetic gene clusters, which suggests a potential application to synthesize new biomolecules. Another paper combined high-throughput culturomics and mass-spectroscopy screening to successfully isolate only the second phototrophic member of the phylum *Gemmatimonadota*, “*Gemmatimonas groenlandica*”, from a stream in Greenland (Zeng et al.). Although genes encoding the photosynthetic apparatus are closely related to that of *Gemmatimonas phototrophica* strain AP64^T^, the new species is distinct based on average nucleic acid identity, pigment composition and absorption spectra, and high oxygen tolerance.

One significant driver of research in extremophiles is biotechnology, given their well-recognized roles as sources of new biomolecules and applications. Included in this volume, the review by Sysoev et al. provides an overview of culture-independent methods, including sequence-based metagenomics and single-cell genomics, for studying enzymes from extremophiles, with a focus on prokaryotes. Additionally, the authors provide a comprehensive list of extremozymes discovered *via* metagenomics and single-cell genomics, which is a useful resource for researchers both in academia and industry. Extremophilic viruses are also rich sources of commercially valuable enzymes, particularly nucleic acid-modifying enzymes. One such example is an unusual DNA polymerase A enzyme from an uncultivated, thermophilic virus that has been developed commercially as a high-affinity, thermophilic reverse-transcriptase (Schoenfeld et al., 2013). In this Research Topic, this *polA* gene and related genes were shown to be encoded by a new family and genus of thermophilic viruses, the genus *Pyrovirus*, that is inferred to infect diverse members of the *Aquificota* based on spacers within clustered regularly interspaced short palindromic repeats (Palmer et al.). The four species of *Pyrovirus* proposed based on complete or near-complete genomes is the first study of viruses infecting *Aquificota*, despite the dominance of *Aquificota* in many terrestrial and marine thermal systems.

## Adaptation to extreme conditions, environmental role and metabolic functions of extremophiles

Mechanisms evolved by extremophiles not just to survive, but to thrive in inhospitable environments are fascinating. Many specialized mechanisms enabling growth under extreme conditions have already been studied while others remain elusive (Coker, 2019; Schmid et al., 2020). Some of the manuscripts in this volume expand on such strategies.

Three papers in the Research Topic focused on energy conservation, central carbon metabolism, and piezophily of thermophiles. The paper by Gavrilov et al. focused on mechanisms used by the thermophile *Carboxydothermus ferrireducens* to use extracellular amorphous ferrihydrite as a terminal electron acceptor for anaerobic respiration, producing large magnetite crystals. Genome sequencing combined with RNA-Seq and proteomic data derived from cultures using different electron acceptors were used to identify three constitutive c-type multiheme cytochromes, including OhmA, which was strongly bound to extracellular magnetite. Another paper by Thomas et al., focused on carbon metabolism in the thermophile *Thermoflexus hugenholtzii* JAD2^T^, the only cultured representative of the *Chloroflexota* order *Thermoflexales*. Despite its chemoheterotrophic metabolism, *T. hugenholtzii* does not grow on any defined carbon sources, obscuring our understanding of its metabolism. Comparative genomics of the *T. hugenholtzii* genome with eight related MAGs from geothermal springs revealed a high abundance and strong conservation of peptidases among three species clusters, suggesting a conserved proteolytic metabolism. Exometabolomics and ^13^C metabolic probing studies confirmed this metabolism for *T. hugenholtzii*. The metabolic probing confirmed glycolysis, tricarboxylic acid (TCA), and oxidative pentose-phosphate pathways, yet glycolysis and the TCA cycle were uncoupled. Microorganisms living at great depths in the sea must cope with variations in hydrostatic pressure. The classical stress response of piezophiles to changes in hydrostatic pressure consists of up- and down-regulation of several genes. Herein, Moalic et al. suggest that *Thermococcus piezophilus*, a piezohyperthermophilic archaeon with the widest range of tolerance to high pressure known so far, modulates its energy-conservation process by means of the control of the master transcriptional regulator SurR under non-optimal pressures conditions.

Two additional contributions focused on acidophily and halophily. Genome comparisons by Cortez et al. on bacterial acidophiles affiliated with different phyla and their neutrophilic counterparts demonstrated a correlation between decreases in genome size and adaptation to low-pH ecosystems. This genome streamlining of acidophilic bacteria is mainly due to reduction of average protein size and gene loss, with an unexpected constant number of paralogs compared with studies that claim a relatively lower paralog frequency for other streamlined microorganisms (Giovannoni et al., 2005; Swan et al., 2013). According to these authors, other potential mechanisms for acid resistance might be the enrichment in cytoplasmic membrane proteins and those involved in energy conservation, DNA repair, and biofilm formation. The paper by Durán-Viseras et al. uses a culturomics approach to isolate new haloarchaea from the Odiel Saltmarsh, Spain, previously revealed to contain a high proportion of sequences not related to previously cultivated taxa (Vera-Gargallo and Ventosa, 2018). Their approach resulted in the isolation and description of three new species: *Halomicroarcula rubra, Halomicroarcula nitratireducens*, and *Halomicroarcula salinisoli*. Ensuing genomic analysis revealed the unexpected identification of complete pathways for the biosynthesis of the compatible solutes trehalose and glycine betaine, not identified before in any other haloarchaea. Although they use a salt-in strategy, this finding might suggest alternative osmoadaptation strategies within this group.

## Phylogeny, evolution, classification, and biodiversity of extremophiles

A heated debate exists on the conditions of the environment where the last universal common ancestor (LUCA) of extant life could have inhabited. Although it is not yet clear if the LUCA was an extremophile (i.e., hyperthermophile), the scientific community agrees that extremophily emerged early in the diversification of prokaryotes (Catchpole and Forterre, 2019). Evolution of the thermoacidophilic archaeal family *Sulfolobaceae* has been a focus of research into this topic. The study by Banerjee et al. revealed a non-symmetric genome evolution within this family, with some species undergoing genome expansion whereas there was gene loss in others, even considering that they share a similar niche. They also detailed a high level of conservation for most of the autotropic pathways across this family.

In the genomic era, systematics has moved forward from gene-based phylogenies to genome-based classifications (Rosselló-Móra and Whitman, 2019; Parks et al., 2022). Thus, taxonomic arrangements supported by whole genome data analysis spanning several groups of extremophiles are proposed through this volume. The research conducted by de la Haba et al. sought to solve a well-known issue concerning the taxonomic status of the partially overlapping and contemporaneously described archaeal genera *Natrinema* and *Haloterrigena* (Minegishi et al., 2010; Papke et al., 2011). Phylogenomic analysis and the use of Overall Genome Relatedness Indexes allowed the authors to clearly differentiate the two genera, and justified transfer several species from *Haloterrigena* to *Natrinema*. In the domain *Bacteria*, the article by Park et al. proposed an average amino-acid identity (AAI) cut-off value of 63.43 ± 0.01% to delineate genera within the *Desulfovibrionaceae*, following up on recent rearrangements in the family based on a phylogenomic approach (Galushko and Kuever, 2020; Waite et al., 2020). As a result, two new genera (i.e., “*Alkalidesulfovibrio”* and “*Salidesulfovibrio”*), and one not yet validly published genus name (“*Psychrodesulfovibrio*”) were proposed. On the other hand, Ramírez-Duran et al. revised the taxonomy of the genus *Saccharomonospora* using a combined approach based on 16S rRNA gene and core-genome sequence phylogenies as well as comparative genomics. Additionally, this article also identifies a wide variety of biosynthetic gene clusters, thus uncovering the potential biotechnological applications of this actinobacterial genus to produce unknown bioactive molecules.

The exploration of natural microbial communities inhabiting extreme environments was the focus of several studies in this Research Topic. These types of studies remain vibrant because they provide insights into the ecology of microorganisms in their natural habitats. As a first example, Liu et al. explored hot springs in Conghua, China, focusing on anaerobic ammonium oxidation (anammox). This is an important process of the nitrogen cycle, and anammox bacteria have been studied in a wide variety of environments. However, the distribution, diversity, and abundance of anammox bacteria in hot springs remains under-reported. Diverse putative anammox organisms were identified, especially “*Candidatus* Brocadia”. In another contribution, Narsing Rao et al. combined culture-dependent and -independent approaches to study seven different hot spring in India. These locations were found to harbor novel microbial groups and some of which produced thermo-stable enzymes. Finally, a separate study focused on benthic microbial community dynamics in an acid saline lake, Lake Magic, Australia, by studying 16S rRNA gene amplicons for prokaryotes and internal transcribed spacer amplicons for fungi over an annual cycle (Ghori et al.). The annual cycle included a flooding stage, followed by evapo-concentration of solutes, which led to decreases in bacterial diversity and increases in halotolerant and acid-tolerant taxa, including phylotypes similar to those involved in sulfur cycling.

## New frontiers

Research on extremophiles has traditionally been at the forefront of microbiology, boldly opening up new frontiers in our understanding of life and its limits. The reach and impact of such research is now extending beyond our own planet, with increasing relevance in the cross-disciplinary fields of Astrobiology and Space Microbiology. The study of extreme environments on Earth and their inhabitants is one of the main pillars of research supporting the search for life on Mars, the exooceans of the icy moons of the solar system, and beyond (e.g., Jebbar et al., 2020; Taubner et al., 2020; Changela et al., 2021). Studies of terran extremophiles is also increasingly seen as useful for assisting space exploration in activities such as *in situ* resource utilization (ISRU; Cockell, 2010; Dhami et al., 2013; Changela et al., 2021).

Space itself can be seen as an extreme environment, with a combination of multiple conditions and with a range of effects on microbes that are not yet fully explored. Observed changes in the characteristics of pathogens and conditional pathogens in space, namely increased pathogenicity and virulence, are a significant concern when discussing long-term crewed mission or human presence in space (Simões and Antunes, 2021). The impact of the space environment seems to vary between species, so the need for further studies has been stressed by several authors.

The current volume of our special topic addresses this issue with a study by Su et al., focusing on the conditional pathogen *Stenotrophomonas maltophilia*, an understudied emerging Gram-stain-negative multidrug-resistant species. This paper constitutes the first general analysis of the phenotypic, genomic, transcriptomic, and proteomic changes in this species under simulated microgravity. Results from this investigation showed that microgravity led to an increased growth rate, enhanced biofilm formation, increased swimming motility, and metabolic alterations. This study further increases our understanding of the effects of space conditions on microbial physiology, metabolism, and pathogenicity and may help to provide new ideas for the prevention and treatment of infections in future missions.

Bijlani et al. describe in this article collection the new species *Methylobacterium ajmalii*, isolated from the International Space Station as part of an on-going microbial tracking experiment that retrieved a few strains from the family *Methylobacteriaceae* (Checinska Sielaff et al., 2019). In addition to the full characterization and description of this new species of *Methylobacterium* using polyphasic taxonomy, the paper further analyses whole genome sequencing data with identification of some specific features with potential relevance for biotechnology.

Another important link between microbiology and space exploration is the study of persistent species in spacecraft assembly cleanrooms. Despite their inhospitable conditions and intense precautions taken within these facilities, different types of microbes are known to persist and are a major source of concern regarding planetary protection measures focused on preventing potential biological contamination of other parts of the solar system (e.g., Rettberg et al., 2019). Within such studies, fungi are frequently overlooked despite the fact that they are known for their resilience and might pose a threat to closed habitats (biocorrosion) as well as their immunocompromised occupants (pathogenicity).

In this sense, Blachowicz et al. deal with isolation and characterization of rare mycobiome associated with spacecraft assembly cleanrooms. The study aimed to help address two current gaps in understanding fungi populations under such settings. On one hand, the majority of previous reports were based on cultivation-based approaches, while culture-independent mycobiome analyses were scarce. On the other, the authors highlighted the need for more efficient cultivation methods when analyzing cleanrooms, particularly as isolation is essential for thorough analysis and characterization of fungi. Metagenomic reads were dominated by *Ascomycota* and *Basidiomycota*, with significant differences between distant locations, and the detection of several potential novel species. This paper is presented as a first step toward characterizing cultivable and viable fungal populations in cleanrooms to assess fungal potential as biocontaminants during interplanetary exploration, in addition to other cleanroom settings, such as intensive care units, operating rooms, or in the semiconducting and pharmaceutical industries.

Schultzhaus et al. also focused on fungi with an eye toward extreme conditions relevant to space exploration, namely ionizing radiation. By studying the phenotypic and transcriptomic responses of the radioresistant yeast *Exophiala dermatitidis* to three different radiation sources (i.e., protons, deuterons, and α-particles), a common theme of induction of DNA repair and DNA replication genes and repression of ribosome and protein synthesis emerged. Yet, differences also emerged, most notably that particle irradiation resulted in greater changes in gene expression and an upregulation of genes involved in autophagy and protein catabolism.

This collection contains a manuscript by Wood et al. that highlights concerns about inconsistent results of different short-read metagenomic pipelines to assess the microbial diversity of the aforementioned cleanrooms. Although all four used pipelines detected extremophiles and spore-forming bacteria, they did not yield the same microbial profile based on the same raw dataset, which raises doubts of the reliability of using a single pipeline. The paper also presents a roadmap aimed at validating future studies on planetary protection-related microorganisms.

## Final remarks

As evidenced by the success of this Research Topic, after over 50 years of research focused on extreme environments, extremophiles are still the subject of enormous interest for the scientific community and for biotechnology. The more we learn about extremophiles, the more fascinating they become, and each new discovery reveals more of their secrets, but also opens up new questions. After all, they are one of the most ancient dwellers on Earth and they will certainly remain here, and possibly beyond our planet, once all of us are gone.

## Author contributions

RH, AA, and BH wrote the first draft of the editorial article, and they also reviewed and approved the final version of the manuscript.

## Conflict of interest

The authors declare that the research was conducted in the absence of any commercial or financial relationships that could be construed as a potential conflict of interest.

## Publisher's note

All claims expressed in this article are solely those of the authors and do not necessarily represent those of their affiliated organizations, or those of the publisher, the editors and the reviewers. Any product that may be evaluated in this article, or claim that may be made by its manufacturer, is not guaranteed or endorsed by the publisher.
